# In Vitro Propagation and Variation of Antioxidant Properties in Micropropagated *Vaccinium* Berry Plants—A Review

**DOI:** 10.3390/molecules25040788

**Published:** 2020-02-12

**Authors:** Samir C. Debnath, Juran C. Goyali

**Affiliations:** 1 St. John’s Research and Development Centre, Agriculture and Agri-Food Canada, St. John’s, Bldg. 25, 308 Brookfield Road, St. John’s, NL A1E 0B2, Canada; 2 Department of Biology, Memorial University of Newfoundland, 232 Elizabeth Avenue, St. John’s, NL A1B 3X9, Canada; juran.goyali@mun.ca

**Keywords:** blueberry propagation, tissue culture, stem cutting, phenolic content, antioxidant activity

## Abstract

The berry crops in genus *Vacciniun* L. are the richest sources of antioxidant metabolites which have high potential to reduce the incidence of several degenerative diseases. In vitro propagation or micropropagation has been attractive to researchers for its incredible potential for mass production of a selected genotype in a short time, all year round. Propagation techniques affect the antioxidant activity in fruits and leaves. Total antioxidant activity was higher in the fruit of in vitro propagated plants compare to the plants grown ex vivo. This review provides critical information for better understanding the micropropagation and conventional propagation methods, and their effects on antioxidant properties and morphological differentiation in *Vaccinium* species, and fills an existing gap in the literature.

## 1. Introduction

The genus *Vaccinium* consists of about 450 species majority of which are widely spread on mountain slopes in the tropics, with the balance being distributed in subtropical, temperate and boreal regions of the northern hemisphere [1,2]. Commercial production of *Vaccinium* berry fruits comes mainly from the species of sections *Cyanococcus* (cluster-fruited blueberries), *Oxycoccus* (cranberries), *Vitis-idea* (lingonberries) and *Myrtillus* (bilberries) [2]. *Vaccinium* berries are characterized by fleshy small to medium-sized fruits with high levels of antioxidant compounds (phenolics, flavonoids, tannins), fruit colorants (anthocyanins and carotenoids), vitamins (ascorbic acid) and minerals [3]. Those fruits are widely renowned for their health benefits, reportedly due to their potent bioactive phenolic compounds which may interact (additively or synergistically) to ameliorate human health conditions [4]. Many *Vaccinium* species are utilized as medicinal plants and ornamental landscape ground cover [5]. The high phenolic compounds having strong antioxidative properties of *Vaccinium* berries are linked to the prevention of several chronic and degenerative diseases including cancer and cardiovascular disorders [6]. The principle function of those antioxidants is to stop or delay the oxidation of other molecules through inhibiting the initiation or propagation of oxidizing chain reactions by free radicals.

Blueberries (*Vaccinium* L. spp.) are popular around the world as a ‘Super fruit’ due to their nutritional value, elevated levels of bioactive phenolic molecules and excellent sensory evaluation [7]. The essential nutritional components include carbohydrate (15.3%), protein (0.7%), dietary fibres (1.5%), fat (0.5%) and water (85%) [2]. Ripe blueberries have 3.5% cellulose and 0.7% pectin [8]. Ripe blueberries have much higher quantity of sugars than leaves, and the most important sugars in fruits are glucose, fructose and galactose [9]. In addition to these essential nutrients, these berries contain a wide range of organic acids, non-nutritive phytosterols such as sitosterol and stigmasterol; and antioxidant phenolic molecules such as phenolic acids, flavonols, flavanols, anthocyanins, proanthocyanidins and ellagitannins (Table 1) [3,10,11,12]. Cranberries ranked first in polyphenol content among the commonly consumed fruits in the North America which relate to high antioxidant activity [13]. Consumption of lingonberries and bilberries has been proved in preventing human cancer which are ascribed to high level of phenolic and anthocyanin compounds [14,15]. Other beneficial phytochemicals including proanthocyanidins, essential minerals, fatty acids, dietary fibre and provitamin A, vitamins C and B-complex are present in higher level in cranberry, lingonberries and bilberries [12,16,17]. Lingonberries are also rich in potassium, calcium, magnesium and phosphorus.

Blueberries are the most widespread and well-known fruits among the commercially important berries of *Vaccinium* species. Although many species of blueberries are native to North America, several of them especially highbush (*V. corymbosum* L.), lowbush (*V. angustifolium* Ait.) and rabbiteye (*V. ashe* Reade) blueberries are commercially cultivated in many countries in Europe, South America, Asia, Australia and New Zealand [37,38]. Blueberry has significant contribution in the fruit industries in Canada and USA. In 2018, Canada produced about 149 thousand MT blueberries, with exports valued at over 360.8 million USD [39], and that was 275 thousand MT in USA which valued total 820.2 million USD [40].

Domestication of the North American indigenous lowbush blueberry also known as wild blueberry has been started in 1961 [41]. However, extensive plantings have not taken place in this continent because of the slow establishment and lack of rhizome production from stem cuttings (SC) which are generally used as propagation materials [42]. Wild blueberries are mostly harvested from naturally grown fields in cold, harsh winter areas in boreal forests, bogs and barrens in Maine in USA and the Atlantic Provinces and Quebec in Canada which is the largest lowbush blueberry producing area in the world [43].

Blueberries and lingonberries are naturally reproduced both sexually from seed and clonally through an extensive underground rhizome system. In general, they are propagated in nurseries using stem or rhizome as starting materials which is easy but time consuming for large scale multiplication. Seeds are used in limited scale as they do not maintain trueness-to-type of donor plants. Traditionally, cranberries are vegetatively propagated with runner cuttings to achieve genetically identical offspring and to preserve advantageous characteristics. However, conventional methods face similar disadvantages as for blueberries and lingonberries [44]. The tissue culture of *Vaccinium* berry plants can be obtained either from node sections or from leaves [45,46]. Cloning by micropropagation is a more demanding and effective method for improving existing wild berry fields as well as for establishing a new farm due to its incredible potential to produce numerous desirable new clones from a single source plant in a short time, all year round [44,47]. Recently, micropropagation has been attractive to researchers for its potential for improving biochemical properties in berry crops including blueberry, lingonberry and strawberry. Micropropagated berries have higher level of phenolic compounds which act as antioxidants in human body. This review provides critical information for better understanding the micropropagation and conventional propagation methods and their effects on morphology and antioxidant properties in blueberries, cranberries and lingonberries, and fills an existing gap in the literature.

## 2. Health Benefits of *Vaccinium* Berry Crops

In the human body, the free radicals cause oxidative damage to the essential molecules like lipids, proteins and nucleic acids. Thus, those radicals are involved in the initial phase of several chronic diseases such as cancer and cardiovascular diseases. In vitro and ex vivo pharmaceutical research has conceded a great deal of information on the bioactivity of blueberry against multiple stages of carcinogenesis, also called oncogenesis or tumorigenesis, and the ability in treatment of several degenerative diseases (Table 2) [3,48,49]. Blueberries are well-known for its anticancer, anti-inflammatory and anti-diabetic properties [50,51]. Fruits or leaves of highbush, lowbush and rabbiteye blueberries induce apoptosis in carcinogenic cells in vitro of various kinds of cancer such as blood [52], breast [15,49], colon [50,53], liver and prostate [54,55,56] cancer, and thus it is believed that blueberry can help preventing human body from those cancer causing diseases.

Wild blueberry extracts reduce the occurrence of ageing related diseases [66,91]. Diets supplemented with 2% blueberry have distinct biological effects on neuronal function and behaviour in aging animals which may be due to the effects of the individual classes of tannins in different regions of the brain [58]. The blueberry products reduce high blood pressure, blood cholesterol and thus prevent cardiovascular and atherosclerosis risks in human body [57,67,68,92]. Daily blueberry consumption improves arterial stiffness in postmenopausal women with pre-stage and stage 1-hypertension [59].

Blueberries exhibit anti-diabetic properties by protecting pancreatic β-cells from glucose-induced oxidative stress [60,69]. A survey has identified Canadian lowbush blueberry as highly recommended fruits by traditional practitioners and Cree Elders of Eeyou Istchee in Quebec for treatment of diabetic symptoms and complications [70,93]. Consumption of European blueberry (bilberry) improves night-time visual acuity in obese mice [87]. It enhances blood and oxygen circulation delivery to the eyes and scavenges free radicals and thus protects against the development of glaucoma, cataract and macular degeneration in mice and humans [88,89,94].

Proanthocyanidins, anthocyanins, and flavonols in blueberries are beneficial in bone protection [95]. Blueberry juice has positive effect to treatment of juvenile idiopathic arthritis [61]. Daily consumption of whole blueberries reduces pain, stiffness and difficulty to perform daily activities, an improved normal walking paced gait performance and would therefore improve quality of life in individuals with symptomatic knee osteoarthritis [62]. Blueberry anthocyanins have been used for several therapeutic purposes including the treatment of fibrocystic disease, vision disorders, radiation-induced cell death [63,90]. A-type proanthocyanidins found in wild blueberry possess antiadhesion and antiproliferation properties for microorganism which help in preventing bacterial infections, especially in the urinary tract [56,96]. Leaf extract of highbush blueberry has significant antibacterial activity against *Salmonella typhymurium* and *Enterococcus faecalis* [64]. The consumption of wild blueberry powder supplements increases a diet-induced ex vivo serum antioxidant status in human body [97]. The extract from leaves, the main waste products in blueberry harvesting as well as in processing industries, inhibits the Hepatitis C virus expression [65]. Consequently, blueberries prevent human health from several chronic diseases.

The cranberry is considered as medicinal fruits and the effects of cranberry products on human health have focused principally on urinary tract infection and cardiovascular disorders [3,35]. Cranberries (juice, concentrated powders, capsule formulations, and tablets) having high-level proanthocyanidins A can prevent recurrence of urinary tract infections by reducing adhesion of *Escherichia coli* to uroepithelial cells which could lower the use of antibiotic treatment and the consequent development of resistance to these drugs [82,83]. Polyphenol rich cranberries contribute in reducing the risk of cardiovascular disease by increasing the resistance of low-density lipoproteins to oxidation, inhibiting platelet aggregation, reducing blood pressure and via other anti-thrombotic mechanisms [78,84]. They have also shown therapeutic activities against breast cancer [85].

Lingonberries have been found to be one of the top competitors with the highest phenolic and proanthocyanidin contents and antioxidant activity among *Vaccinium* berries [31,33] which exhibit significant anti-genotoxic, anti-mutagenic and apoptotic effects on human leukemia and colorectal cancer cells in vitro [48,72,74]. These berries reduce liver inflammation and exert anti-obesity property by decreasing body fat in high-fat diet mice [75,76]. Bilberries have been used in the traditional medicine internally (as tea or liqueur) for treatment of disorders of the gastrointestinal tract and diabetes [33]. Their anthocyanins demonstrated anticancer properties by inhibiting cancer cell proliferation and by acting as cell anti-invasive factors and chemo-inhibitors [15].

## 3. Phenolics in *Vaccinium* Berries

*Vaccinium* berries are mostly popular for their antioxidant phytochemicals especially phenolic metabolites that play significant role not only in plant defence mechanism, but also in human health benefits. The largest category of phytochemicals, polyphenolic compounds, are widely distributed in the leaves, fruits, seeds and flowers. Their structures range from simple moieties containing a single hydroxylated aromatic ring to highly complex polymeric compounds. The most of plant phenolics are classified into flavonoids and non-flavonoids [98]. The chemical structure of flavonoid compounds is based on two aromatic benzoic rings connected by a bridge consisting of three carbons (C_6_-C_3_-C_6_) [99]. Flavonoids are compounds of low molecular weight usually bound to sugar molecules. They are divided into anthocyanins and anthoxanthins. Anthocyanins are red, blue and purple pigment molecules, and anthoxanthins that include flavonols, flavones, flavanols and isoflavones, are colourless or white to yellow molecules [100]. Non-flavonoids include phenolic acids (hydroxybenzoic C_6_-C_1_ and hydroxycinnamic C_6_-C_3_), lignans (C_6_-C_3_)_2_ and stilbenes (C_6_-C_2_-C_6_). Phenolic acids and flavonoids account for 60% and 30% of total dietary plant phytochemicals, respectively [99]. Other two non-flavonoid subclasses are tannin and lignin which are the polymers of particular phenolic compound and have high molecular weight with unique structure [98]. Condensed tannins, a subclass of flavonoids, are polymers of catechins and epicatechins and found mainly in fruits, grains and legumes. The biosynthetic pathways of phenolic substances in plants are predominantly controlled by endogenous processes during developmental differentiation [101]. Plant phenolics are synthesized from a limited pool of biosynthetic precursors such as pyruvate, acetate, acetyl coenzyme A (CoA), malonyl CoA and a few amino acids [102] following pentose phosphate, shikimate and phenylpropanoid metabolism pathways [103].

Certain derivatives of hydroxybenzoic or hydroxycinnamic acids such as chlorogenic, caffeic, *p*-coumaric, ellagic and vanillic acids are widely distributed in leaves and fruits of *Vaccinium* berries as natural antioxidants [32,104,105,106]. Important group of flavonoids found in blueberry, cranberry, lingonberry and bilberry are flavonols (quercetin derivatives), anthocyanidins, proanthocyanidins, catechins and their glycosides [31,106,107]. Among over 300 different anthocyanidins found in plants, cyanidin, delphinidin, petunidin, peonidin and malvidin derivatives are most common in those berries [21,31,108]. Anthocyanins, glycosidic forms of anthocyanidins, are major pigments in dark and bright colour fruits such as blueberry, cranberry, lingonberry and bilberry. Proanthocyanidins, which differ from other phenolic compounds by their polymeric structure, are predominantly distributed in blueberry at green stages and leaves [109,110]. Proanthocyanidins can bind strongly with carbohydrates and proteins, and act as strong free radical scavengers. Those are believed to be at least 15 to 25 times stronger in antioxidant capacity compared to vitamin E, and demonstrate a wide range of pharmacological activity [111].

Phenolic and flavonoid compounds have significant contribution in plant defence mechanisms, fruit development and seed dispersal. Flavonoids especially proanthocyanidins or condense tannins are found in immature fruits where their astringency and bitterness help to deter frugivores from consuming fruit before they are ripe [112]. The phenolic compounds such as lignin, cutin, suberin are the integral parts of the cell-wall of plants serving as mechanical support [113]. Those metabolites are accumulated to defend plants against infection [114], mechanical wounding [115], nutritional stress [116], cold stress [117,118], light and heat stress [119]. Longer photoperiod (24 h) enhanced synthesis of anthocyanin, its derivatives and chlorogenic acid compared with a shorter photoperiod (12 h) in *V. myrtillus* grown in Finland [120]. The constitutive phenolics of wild blueberry exhibited variable resistance to aphid attack within and between blueberry populations [121]. The infestation of herbivorous arthropods, mealybug and mite, influenced accumulation of the phenolic compounds in orchid and strawberry leaves as a defence mechanisms [122]. An important function of anthocyanins together with flavones and flavonols is pigmentation of flowers and fruits [123] which attract insects and birds to the plant for pollination and seed dispersal [124]. Furthermore, deficiency of iron, phosphorus and nitrogen in soil, drought conditions, over application of herbicides can also trigger the production of phenolic compounds in plants as a means of tolerance [108,119,125]. Phenolic substances influence the competitive phenomenon called ‘allelopathy’ among the plants and weeds [126,127]. In addition to the familiar volatile terpenoids, simple phenols such as hydroxybenzoic acids and hydroxycinnamic acids affect the growth and development of agricultural and biological system [126].

## 4. Propagation of *Vaccinium* Berries

Due to awareness of the health-promoting properties of *Vaccinium* berries, the market demand and growing areas specially for developed cultivars of blueberries and cranberries have been dramatically increased during the last two decades in Canada, China and Turkey [20,39,128]. Numerous planting materials are required to cope with the high demand for those berries to establish new farms. Although lingonberry, cranberry and lowbush blueberry produced from wild stand need minimum cultivation practice, number of cultivated farms is increasing. In a naturally grown commercial field of lowbush blueberry, there are many bare spots raised from herbicide application or mechanical scalping which rendered for low production. To cover up those incomplete areas, planting materials propagated through conventional SC propagation are generally used. Stem or rhizome cutting propagation is easy but time consuming for large scale multiplication. Tissue culture technology is becoming attractive propagation method to the nursery owners as well as to the berry producers due to its fast spreading capacity through producing a great number of stems, rhizomes (underground stems) and branches [32,129,130]. Different propagation methods are discussed below:

### 4.1. Sexual Propagation

Lowbush blueberries are generally self-incompatible, but a significantly higher incidence of self-fertility has also been reported in several genotypes [131,132]. True seeds developed from fertilized ovules in cross-pollinated flowers of blueberry and cranberry are used as a means of sexual propagation. Lingonberries are self-pollinating species, but cross pollination produces larger fruits. Cross pollination of these berry flowers occurs mainly via insect pollinators like rented honeybees and native bees which are thought to be attracted to the plants by the vibrant colour and aromatic scent of the flowers [124]. Genetic materials of two parents are combined in a progeny of sexual propagation having a new genetic makeup which is not identical to the mother plant. Although sexual propagation is easy and numerous seedlings can be grown from a single source plant, the seedling progenies produce <50% fruits of their parental clones of lowbush blueberries [133]. In sexual propagation, lowbush blueberry plants usually flower and develop rhizomes 3–4 years after seed germination.

### 4.2. Asexual Propagation

Asexual reproduction occurs naturally in wild blueberries when their rhizomes are cut or killed by fire, shading, burrowing, or frost action [134]. Other *Vaccinium* berries are vegetatively propagated with stem or root cuttings and by micropropagation which preserve the desired genetic characteristics of parent materials and achieve rapid fruit bearing capability [45].

#### 4.2.1. Propagation by Stem Cutting

**Figure 1:**
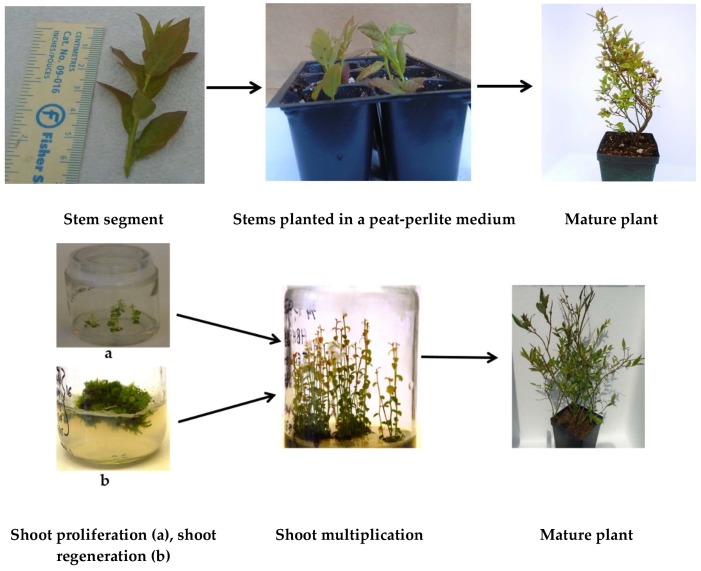
Softwood cutting propagation (upper panel) and micropropagation (lower panel) of lowbush blueberry [30,130].

Vegetative propagation of *Vaccinium* species has long been successfully practiced using the nodal segments of softwood, semi-hardwood, hardwood stems, single node, division of sub-terrestrial rhizomes or even leaf-bud cuttings as propagules to reproduce genetically identical plants called clones which preserve the genetic structure and uniformity of source plant. The most widespread practice is softwood cutting using young shoots or shoot tips containing meristem (Figure 1). About 4–6 cm long shoot tips are clipped from mother plant and planted in potting soil could be supplemented with growth hormones or in field directly [135]. The SCs grow shoots and develop adventitious roots (Figure 1) within several weeks with maintenance of proper soil fertility, temperature, humidity and light intensity and duration. The alternative to softwood cuttings is hardwood cuttings, which refers to cuttings taken once the plant tissue becomes woody, typically at the dormant stage of plants. Semi-hardwood and rhizome segments are clipped from the matured plants and place in soil media for rooting. The SC propagation is time consuming for large scale multiplication of *Vaccinium* species, since limited number of propagules can be prepared from a single source plant. Another difficulty of conventional propagation is that SCs have limited potentiality to develop new and subsequent rhizomes which slow down the spreading tendency, and they commonly face challenges in rooting capacity [136,137]. Since the *Vaccinium* berry crops are heterogeneous species due to inclusion of numerous wild clones with divergent clonal characteristics, it is a crucial problem for commercial propagation and establishment of selected clones. As demand increases for these fruits from industry and global consumers, the importance of commercial propagation increases as well. The shortcomings of SC propagation can be overcome by using in vitro techniques [138] which could fulfil the world demand of blueberry supply.

#### 4.2.2. In Vitro Propagation or Micropropagation.

In vitro propagation, also called micropropagation, is carried out in control environments using cells, tissues or organs of a plant as explants. The explants are grown on an artificial medium consisting of water, macronutrients and micronutrients, some carbon source (usually carbohydrates in the form of sucrose or glucose), vitamins, growth regulators (auxins, cytokinins and gibberellins) and a chelating agent (in the case of solid medium). Under aseptic conditions, all those media components act together to supply optimum nutrients that allow plant growth [46]. The entire procedure is carried out in aseptic condition and growth media are changed regularly to replenish elements to continue tissue growth. In vitro propagation is operated based on enhanced axillary bud proliferation and on the ability of plant cells to differentiate and develop new meristematic centres that are capable of regenerating fully normal plants [139]. Regeneration of meristem or shoot or root is carried out through three different morphogenic pathways [44]: (i) axillary shoot proliferation from pre-existing apical or axillary buds, (ii) organogenesis through formation of unipolar organ or shoot regeneration and (iii) somatic embryogenesis through development of bipolar structures, somatic embryos with both root and shoot meristems [140]. The choice of starting material or explant in tissue culture determines the path through which the explant will go to produce new shoots and plants.

Plant regeneration through tissue culture relies on two basic concepts: totipotency and developmental plasticity. Totipotency is the ability of a cell to differentiate, proliferate and subsequently turn into a mature plant under appropriate culture conditions in a hormone-dependent manner [141]. In general, totipotency is a characteristic of the cells in young tissues and meristems, but it can also be exhibited by some differentiated cells [44]. Although, a whole plant could be regenerated solely from one cell, practically it is a challenging process. When an explant is provided with correct stimulus hormone(s) and appropriate environments, it develops into a plant identical to the source plant or clone. Tissue culture can rapidly and aseptically produce large amount of plant material, while selecting for and cloning superior germplasms that are disease-resistant and produce elevated levels of vegetative growth. The tissue culture technique is a very efficient propagation method for *Vaccinium* plants.

Major advantage of micropropagation is that it ensures rapid and continuous supply of mass production of healthy, genetically identical and pathogen-free plants all the year round [142]. It is an invaluable aid in the multiplication of male sterile, fertility maintainer and restorer lines. In breeding programs for perennials, micropropagation can accelerate the breeding process by in vitro selection and in a replicated trial of new releases [44]. In vitro technology also offers several advantages over naturally grown plants in producing bioactive compounds [143,144] such as (i) production conditions can be optimized and controlled to get desired content of pure product; (ii) biological factors such as microorganisms, insects and climatic and geographic conditions cannot affect the production of secondary metabolites; and (iii) automated control of cell growth would reduce labour costs for bioactive compound production. However, micropropagation is a complex procedure and requires sophisticated facilities which involve expensive machinery and reagents. It demands highly trained and skilled labours in handling and maintenance of cultures. Tissue culture procedure, media composition and growth regulators varied depending on the plant species and even on different genotypes of the same species [145], which also increases the expense of the method. Rooting of micro-cuttings in vitro is expensive and can even double the price of the cutting [146]. Sometimes plants do not produce trueness-to-type regenerants which limit the goal of commercial micropropagation.

In vitro culture of blueberries was initiated in early 70′s by Barker and Collins [147] who grew rhizome pieces on White’s medium [148] without adding growth regulators. Boxus [149] and Anderson [150] were the founders for commercial micropropagation of berry crops. Although tissue culture for highbush and half-high blueberries has been routinely used for more than thirty years [151], micropropagation for lowbush blueberry is in developing stages. The first callus formation was induced in vitro in lowbush blueberry using stem internodes by Nickerson and Hall [152] on Murashige and Skoog medium [153] supplemented with growth hormone 2,4-dichlorophenoxyacetic acid (2,4-D) (Table 3). After two years, Nickerson [154] induced shoots from blueberry seedling explants and the author developed callus in same genotypes using fruit explant [155]. Nowadays tissue culture techniques are practiced through axillary shoot proliferation and adventitious shoot generation using semisolid and liquid media for lowbush blueberries [138,156,157,158,159] and their interspecific hybrids half-high [160,161,162] blueberries (Table 3). The most recent progress in blueberry micropropagation is development of somatic embryogenesis [163] in blueberry cultivars and utilization of automated bioreactor systems with liquid media for multiplication of micropropagules of lowbush and half-high blueberries derived through either shoot proliferation or adventitious shoot regeneration [162,164,165]. Micropropagation of cranberry and lingonberry species from axillary meristems as well as shoot organogenesis from leaf explants with different plant growth regulators have been well-established [135,139,166]. Bioreactor system is cost effective for commercial propagation. However, liquid culture is generally limited by low oxygen content and hyperhydricity in regenerants [164,167]. Another problem in micropropagation for blueberry with shoot explant is the formation of unwanted callus at the base of the explants and the occurrence of spontaneous adventitious shoots [168,169]. Appropriate growth hormone specially auxin and optimum auxin cytokinin ratio help to overcome this problem. Litwińczuk and Wadas [169] reported that using indole-3-butyric acid (IBA) instead of indolyl-3-acetic acid (IAA) and lowering N6-(2-isopentenyl) adenine (2iP) concentration enhanced healthy axillary shoot with relative long internodes and rigid, well-developed leaves in highbush blueberry and suppressed base-adjoin unexpected shoots which were thin and fragile, mostly vitrified with short internodes, smaller and unfolded leaves.

## 5. Micropropagation, Morphology and Antioxidant Phenolic Contents in *Vaccinium* Berries

Although a number of reports are available on morphological variation in micropropagated *Vaccinium* species, few are available for antioxidant properties [30,32,130]. Micropropagation exhibited a remarkable influence on growth habit and morphology of *Vaccinium* berry plants. Micropropagated plants of lowbush blueberry cultivars and wild clones had vigorous growth, taller and greater number of stems with more leaves, and produced larger canopy than the SC plants (Table 4) [130,138,173]. In vitro derived highbush and half-high blueberry plants grow faster with taller and more shoots than SC plants [197,198,199]. Higher number of shoots and rhizomes, taller plants, more leaves were reported in tissue cultured lingonberry and cranberry plants compared to SC counterparts [32,190,193]. Leaf size in blueberry plants was significantly influenced by propagation methods. Litwińczuk et al. [199] reported that tissue culture plants of highbush blueberry produced wider leaves compared to SC ones. Conversely, Brissette et al. [172] found that reversion of matured plants to juvenile state in in vitro culture produced small and round shape leaves. A direct result of residual action of growth hormones especially cytokinins used during in vitro propagation influenced the vegetative growth of micropropagated plants of *Vaccinium* species [47,191,193,200]. However, SC lowbush blueberry plants flowered abundantly, bore significantly higher number of berries, thus apparently yielded better than tissue cultured plants [30,129]. Litwińczuk et al. [199] and Vyas [32] reported similar results in highbush blueberry and lingonberry, respectively. On the contrary, better yield of in vitro derived plants without deteriorating fruit quality was reported in half-high ‘Northblue’ [198] and lingonberry ‘Sanna’ cultivars [201]. Whereas, no difference was found between established field-grown SC and micropropagated plants of half-high and lowbush blueberries for the number of flowers and berry weight per plant [47]. Plants derived from in vitro propagation directed significant amounts of energy into the production of new axillary shoots and rhizomes and were therefore potentially limited by a commitment to vegetative growth that might have restricted the size and weight of fruits [202]. In contrast, SC berry plants showed energy conservation by producing fewer, if any, rhizomes and only one primary shoot thereby allowing bigger size fruit ultimately increased berry weight [200].

Plant cell, tissue and organ cultures appear as viable biotechnological tools for elevating the level of bioactive metabolites in higher plant species. The advantages of micropropagation in several medicinal plants to produce antioxidant metabolites are available to fulfill the high pharmaceutical demands [143,204,205,206]. Although blueberry is one of the highest phenolic containing fruits, application of tissue culture to enhance the antioxidant quality of fruit is in developing stage. Micropropagated lowbush blueberry clones have higher contents of phenolics and flavonoids and their antioxidant activity compared to blueberries developed from conventional SCs, in spite of the fact that both plants were grown under the same environmental conditions in the greenhouse (Table 4) [30,203]. Other *Vaccinium* berries like bilberry and lingonberry have similar trend in phenolic and flavonoid content in micropropagated plants [32,195]. Variation in phytochemical contents was reported by Ghosh et al. [163] between donor blueberry plants and their somatic embryogenesis (SE) regenerated plants. They reported that total phenolic and flavonoid contents were higher in SE-regenerated plants than its SC donor plants in half-high blueberry cultivars. In vitro propagated blueberry and bilberry have higher antioxidant potential compared to SC plants [195,203]. The total DPPH [2,2-diphenyl 1-picrylhydrazyl] radical scavenging capacity was higher in fruit extract of three lingonberry cultivars ‘Regal’, ‘Erntedank’ and ‘Splendor’ derived through node and leaf cultures compared to conventional SC plants [32,202].

The stimulatory role of micropropagation in increasing phenolic content might be because of plant growth regulators used in media on biosynthesis of phenolic compounds through influencing the expression or up-regulation of genes involved in the biosynthetic pathway of secondary metabolites [110,207]. For instance, cytokinin alone or in combination with auxin, gave a significantly increased amount of total phenolics, flavonoids and condensed tannins in *Aloe arborescens* species, in comparison to plant growth regulator-free medium during in vitro propagation through direct shoot proliferation [163,208]. The level of transcription of the genes in flavonoid biosynthesis pathway encoding phenylalanine ammonia lyase, chalcone synthase, chalcone isomerase and dihydroflavonol reductase, were shown to increase coordinately with cytokine concentration and thereby enhancing the anthocyanin level in *A. thaliana* [209]. On the other hand, auxins regulate the pool size of active cytokinins by promoting cytokinin glucosylation and oxidative breakdown to others [210]. The choice of cytokinin and its concentration in tissue culture makes a difference in the production level of secondary metabolites.

The phenolic and flavonoid contents and their antioxidant activities in blueberries are variable depending on the species, cultivars and varieties, tissues or organs, developmental and maturity stages, growing seasons and locations. The effects of those factors on phenolic content in *Vaccinium* berry plants with respect to micropropagation are discussed.

### 5.1. Genotype Specific Action of Micropropagation for Phenolics and Antioxidant Capacity

Propagation methods influence the capacity of blueberry plants to synthesize polyphenols and flavonoids, and certain genotypes varied in their capacity under different propagation conditions. The wild clone of lowbush blueberry was highly influenced by micropropagation compared to the developed cultivar ‘Fundy’ [30]. In that study, total phenolic, flavonoid, anthocyanin and proanthocyanidin contents in the fruit extract of wild clone ‘QB9C’ was higher in tissue culture plants than in conventional softwood cutting counterparts; whereas none of the phytochemical contents of ‘Fundy’ was changed significantly by propagation methods. Genotype specific tissue culture effect also reported in highbush and half-high blueberry plants. In vitro cultured highbush cultivar ‘Bluegold’ contained higher amount (about twice) of phenolic compounds than the other cultivars’ Duke’, ‘Legacy’, ‘Brigitta’, ‘Elliott’ and ‘Misty’ [25]. Tissue culture regenerated plants of half-high cultivar ‘Chippewa’ contained higher level of total phenolics and flavonoids than their SC donor plants [163]. In the same study, ‘Northblue’ cultivar responded differently to in vitro propagation for synthesizing phenolic compounds. Total phenolic and flavonoid contents were less in the tissue cultured plants of ‘Northblue’ than its donor plants. However, the micropropagated plants of ‘Patriot’ and ‘St. Cloud’ cultivars did not show any significant difference in total phenolic contents from conventionally propagated plants.

Among three cultivars of lingonberry, two cultivars ‘Regal’ and ‘Splendor’ were influenced by micropropagation for their anthocyanin synthesis which resulted higher anthocyanin content in tissue culture plants than SC counterparts [32]. However, leaf tissues of SC ‘Erntedank’ cultivar had higher anthocyanin content than of micropropagated plants. In the same study, SC ‘Regal’ and ‘Splendor’ had higher tannin content than in vitro derived plants whereas, ‘Erntedank’ did not exhibit any different response to the propagation methods for the tannin synthesis.

Genotype-specific response to propagation methods for antioxidant activity have been reported in lingonberry. Micropropagated berries of two cultivars ‘Erntedank’ and ‘Splendor’ exerted higher antioxidant activity than SC plants, whereas both SC and tissue culture plants of ‘Regal’ have similar antioxidative property [32,202].

### 5.2. Tissue Culture Effects on Fruits vs. Leaves for Phenolics

Phenolic and flavonoid compounds are not evenly distributed in all the plant tissues or organs. Those compounds vary considerably among leaves, flowers, fruits or even in the different fruit parts of blueberry species. Compared to fruits, leaves of blueberries contain higher level of phenolic compounds [26,30,71,109,130,211], although anthocyanin content is more in ripe berries. Higher concentration of phenolics, flavonoids, anthocyanins and condensed tannins were reported in leaves of other *Vaccinium* species such as lingonberry, cranberry and bilberry compared to their fruits [32,71,212]. Conversely, Alam et al. [213] reported that mean phenolic content was higher in fruits than in leaves of wild lingonberry populations across Newfoundland and Labrador, Canada.

Propagation methods affects differently the synthesis of phenolic compounds in various plant tissues. The content of total phenolics and other antioxidant metabolites showed different (often opposite) patterns in fruits from leaves. In lowbush blueberries, higher levels of polyphenols were reported in the leaves of SC propagated plants than tissue culture plants [130], while the berries from in vitro propagated plants had higher level polyphenols, flavonoids and anthocyanins than the fruits from of SC plants [30,203]. Similar tendency was reported in other berry species. Leaves from micropropagated lingonberry and strawberry contained significantly less phenolics, anthocyanins and proanthocyanidins compared to conventional SC plants [32,214]. Conversely, berries from tissue culture plants of lingonberry had higher contents of phenolics, flavonoids and proanthocyanidins than SC plants. Whereas, anthocyanin content in SC lingonberries was higher than tissue cultures counter parts [32,202]. Tissue culture methods affect the composition of anthocyanins synthesized in callus formation. Anthocyanin composition and accumulation were simpler and lower in in vitro developed pigmented callus than those present in the fruits and leaves of donor plants of bilberry and blueberry [49,215]. The synthesis of individual phenolic compounds such as quercetin, catechin, epicatechin, *p*-coumaric acid and gallic acid were influenced by micropropagation in fruits of lingonberry cultivars and those were higher in micropropagated plants than SC fruits, whereas, the leaves of tissue culture plants had lower level of those compounds than in leaves of SC counterparts [32].

Antioxidant activities of *Vaccinium* species differ significantly in various plant tissues. Total antioxidant activity measured as DPPH radical scavenging activity of leaves from SC lowbush blueberry plants were higher compared to tissue culture counterparts where antioxidant capacity of micropropagated fruits was higher than softwood cutting berries [30,130]. Vyas et al. [32] reported that DPPH radical scavenging capacity in lingonberry leaves was not affected by the method of propagation whereas in fruits of the tissue culture plants, the antioxidant capacity was ~10% higher than SC plants. Antioxidant capacity was significantly higher in the leaf tissues of several highbush and half-high blueberry cultivars than in fruits of respective genotypes [26]. Comparing with root, the leaves of two highbush blueberry cultivars ‘Legacy’ and ‘Bluegold’ had more than double DPPH radical scavenging capacity [216]. Higher antioxidant capacity in leaves compared to fruits might be due to the higher levels of phenolic and flavonoid contents in leaves. In lingonberry species, propagation methods did not influence the antioxidant capacity in the leaf tissues whereas, micropropagated berries showed higher DPPH radical scavenging capacity than conventionally propagated plants [32].

Phenolic compounds, the most abundant secondary metabolites in higher plants are derived from their common precursor phenylalanine which is produced in plants via the shikimate pathway [119]. Different environmental factors such as low light conditions and lower concentration of nutrients in growing media increase the activity of phenylalanine ammonia lyase enzyme, which is a crucial regulatory factor of phenol metabolic pathway [217]. Under greenhouse condition, prolonged culture of plants could initiate low nutrient stress in the SC plant system which might act as an enhancer of phenolic metabolites in leaves [130]. It was reported that total phenolics and monomeric anthocyanins could be elevated in field grown ripe blueberries and red leaves by applying stress inducing growth regulators [109,218]. In another study, Khalil et al. [219] reported higher phenolic and flavonoid compounds in vitro shoots of stevia (*Stevia rebaudiana)* plant treated with growth regulators compared to the control non-treated one.

### 5.3. Development-Specific Action of Tissue Culture for Phenolics

The synthesis of phenolic and flavonoid compounds varies significantly in relation to the physiological development of fruits, being a result of equilibrium between biosynthesis and further metabolism. Most important control mechanisms in the phenolic metabolism include synthesis and activities of enzymes, location of enzymes, accessibility of precursors and intermediates and integration in the differentiation and development programs [220,221]. The concentration of phenolic compounds is usually higher in young fruit tissues which drops steadily with the advancement of maturity stages, and those rise again at the end of maturation in most of the red, purple or blue fruits such as lingonberry, cranberry and blueberry in which anthocyanin or flavonoid pigments accumulate expressing ripening of berries.

The synthesis of phenolic compounds at different growth and developmental stages of higher plants responds to propagation methods. Makowczyńska et al. [222] reported that the shoots at vegetative stages (3 months old) harvested from in vitro and in vivo propagated black horehound (*Ballota nigra)* plants were found to have a lower level of phenolic and flavonoid compounds than in the shoots at matured flowering stages of same plants.

### 5.4. Seasonal Effect on Micropropagation for Phenolics

The synthesis of phenolics in blueberry is affected by growing season which are rendered to variation in environmental factors such as light, temperature, humidity and precipitation. The influence of micropropagation on the secondary metabolite contents in blueberries varied in respect to the growing seasons. Goyali et al. [30] reported that higher contents of anthocyanin and proanthycyanidin in micropropagated lowbush clone ‘QB9C’ compared to SC plants were exhibited in one growing season (2012) which were not significantly different in the following seasons (2013). In the same study, the developed cultivar ‘Fundy’ exhibited significant difference in flavonoid contents between tissue culture and conventionally propagated plants in one season out of two.

Antioxidant activity of blueberries varies from location to location and from year to year, but this variation is genotype specific. The influence of micropropagation on the antioxidant activities in blueberries differed in one growing season from other. Goyali et al. [30] reported that higher antioxidant capacity of tissue culture wild clone ‘QB9C’ compared to SC plants were exhibited in one growing season (2012) which were similar in next season (2013). The effects of production year and location on phytochemical content and antioxidant activity are dominant and genotype specific.

## 6. Conclusions and Future Direction

*Vaccinium* berry crops including blueberry, cranberry and lingonberry are well-known for their commercial and nutritive values with high antioxidant metabolite contents which have high potential to prevent several degenerative diseases. Despite the high demand of lowbush blueberry due to its health benefits, its major portion is commercially harvested from wild stands and conventionally propagated farms. Although, tissue culture plants have enhanced morphological and biochemical potential in berries, the development of somaclonal variation (in vitro-derived variation) [153] may inhibit acceptance of tissue culture plants for commercial production. Micropropagation of *Vaccinium* berry plants is well-established which could be an alternative tool for improving phytochemicals in these berry crops. Micropropagation influenced the synthesis of phenolic and flavonoid compounds, and their antioxidant activities in blueberries and lingonberries. However, tissue culture effects were specific to genotype, tissue or organ, development and maturity stages and seasonal variations. Tissue culture enhances synthesis of phenolics in one cultivar which may not occurred in another cultivar of same species. Blueberry and lingonberry leaves contain substantially higher levels of polyphenolics, flavonoids and proanthocyanidins than those in the fruits. Leaf tissues respond to the tissue culture in diverged way than the fruits of same plants do for their phytochemical contents. The leaves of conventionally propagated blueberry plants contained phytochemicals in higher level and performed greater antioxidant activity than the leaves of micropropagated plants did. Whereas tissue culture fruits have higher level secondary metabolites compare to SC fruits. Maturity stage plays an important role in phenolic synthesis. The berries at early developmental stages contained higher level of phenolics than matured stages. In case of leaf tissue, matured leaves had higher bioactive phytochemicals and antioxidant potential than the green leaves. Green fruits had significantly higher phenolic and flavonoid content and antioxidant activity compare to semi-ripe and fully ripe berries and those were gradually decreased with the progression of ripening. In contrary, anthocyanin content increased with the advancement of fruit maturity. Growing seasons exhibited significant effect on the total phenolic, flavonoid, anthocyanin and proanthocyanidin contents and antioxidant activity.

The influence of growth regulators used in micropropagation plays an important role in the expression of genes involved the flavonoid biosynthetic pathways. The expression of those genes is up- or down-regulated at different tissues, and various maturity stages of a tissue which causes variation in flavonoid profile at various maturity stages. The gene expression study at different maturity stages of micropropagated blueberry plants will give a clear picture of the changes of phenolic content and its profile variation caused by tissue culture technology. Epigenetic variations especially global DNA methylation have been detected in blueberry species using methylation sensitive amplified polymorphism (MSAP) technique which are triggered by tissue culture. Micropropagated lowbush blueberry plants demonstrated higher global DNA methylation compared to SC plants [223]. DNA methylation are involved in gene expression. Although MSAP technique detects global DNA methylation pattern in blueberry based on the recognition sites of isoschizomer pairs, the methylation status in a specific gene or loci is undermined in MSAP analysis. Bisulfite modification and characterization of the genes involved in metabolite synthesis pathways under tissue culture system will help to better understanding the correlation between DNA methylation and changes in phytochemical synthesis in blueberry plants. Further investigation of individual genes in flavonoid synthesis pathways of micropropagated plants will help to understand the effect of propagation methods on phytochemical content under different maturity stages of *Vaccinium* berry crops.

The increased antioxidant activity and morphological characters including growth of in vitro-propagated *Vaccinium* berry plants should have practical application to the growers for early fruit production and quick establishment of these crops under field and/or greenhouse conditions [200].

## Figures and Tables

**Table 1:** Total phenolic, flavonoid, anthocyanin and proanthocyanin contents in *Vaccinium* berries (mg/100 g fresh weight).

Berry Types	Phenolics	Flavonoids	Anthocyanins	Proanthocyanidins	References
Highbush blueberries	77.0–820.0	155.2–512.3	18.0–249.0	179.8	[11,18,19,20,21,22,23,24,25]
Half-high blueberries	110.0–668.0	161.7–492.1	94.5–310.0	–	[22,26,27,28]
Lowbush blueberries	299.0–840.0	260.0–320.0	59.0–344.0	190.0–331.9	[11,18,20,21,29,30,31]
Rabbiteye blueberries	230.8–929.6	–	12.7–410.0	–	[11,18,19,23]
Lingonberries	489.1–760.0	692.0–1047.0	35.0–708.8	278.8–1294.7	[31,32,33]
Cranberries	328.0–915.0	278.0–751.0	13.0–227.0	11.2–418.8	[31,34,35]
Bilberries	458.0–570.0	374.0–418.0	301.0–393.0	85.5	[21,31,33,36]

**Table 2:** Bioactive compounds in *Vaccinium* berries and their biological properties.

Berry Types	Bioactive Compounds	Biological Properties	References
Highbush blueberries	Polyphenols, anthocyanin, tannins; β-carotene, lutein and zeaxanthin	Anticancer, anti-inflammatory, anti-microbial activities; retard and reverse age-related deficits in behaviour; reduce cardiovascular risks; ameliorate radiation-induced lung injury; retard type II diabetes, juvenile idiopathic arthritis and osteoarthritis.	[3,52,55,57,58,59,60,61,62,63,64,65]
Lowbush blueberries	Phenolics, flavonoids, anthocyanin and proanthocyanidin fractions	Retard liver and prostate cancer; inhibit urinary tract infections; reverse signs of aging; protect brain against ischemia-damage; strengthen blood vessels and arteries; neuroprotective effect.	[48,54,55,56,66,67,68,69,70,71]
Rabbiteye blueberries	Polyphenols, anthocyanin, tannins	Inhibit colon and liver cancer.	[50,53]
Lingonberries	Polyphenols, anthocyanin and proanthocyanidin fractions	Prevent the detrimental metabolic effects induced by high-fat diet; protect kidney against ischemia–reperfusion induced kidney injury; anti-inflammatory, anticarcinogenic, antimicrobial, antiadhesion activities; prevent leukemia and colon cancer.	[31,48,71,72,73,74,75,76,77]
Cranberries	Polyphenols, anthocyanin and proanthocyanidin fractions	Antibacterial, anticarcinogenic activities; reduce cardiovascular risk in patients with metabolic syndrome; protect from diet-induced obesity and insulin resistance; prevent intestinal oxidative stress, inflammation and urinary tract infection.	[3,31,48,73,78,79,80,81,82,83,84,85]
Bilberries	Anthocyanins, flavonols, carotenoid, lutein, and zeaxanthin	Anticarcinogenic; reduce inflammation and progression of chronic hypertension; prevent development of glaucoma, cataract and macular degeneration.	[15,48,49,51,86,87,88,89,90]

**Table 3:** Examples of in vitro propagation of *Vaccinium* species using different basal media and explants.

Species	Media Types ^1^	Micropropagation Via	Explants Used	Rooting In Vitro/Ex Vitro	References
*V. corymbosum* × *V. angustifolium* cv. ‘St. Cloud’, ‘Patriot’, ‘Northblue’, ‘Chippewa	MBM-C	Somatic embryogenesis	Leaf segments	In vitro & ex vitro	[163]
*V. angustifolium* wild clones	MBM-C	Shoot proliferation	Single nodes, axillary buds	Ex vitro	[157]
*V. angustifolium* cv. ‘Fundy’ and wild clones	MBM-C	Shoot proliferation	Shoot tip and segments	Ex vitro	[138,158]
*V. angustifolium* wild clones	MBM-C	Shoot regeneration	Leaf segments	Ex vitro	[158,165]
*V. angustifolium*	WPM	Shoot proliferation	Single node	N/R	[170]
*V. angustifolium*	ANM	Shoot regeneration	Hypocotyl and cotyledons	N/R	[154]
*V. angustifolium*	MSM	Callus formation	Internodes and fruits	N/R	[152,155]
*V. angustifolium*	ZBM	Shoot proliferation	Shoot	Ex vitro	[156]
*V. angustifolium*	ZBM	Shoot proliferation	Young shoot	Ex vitro	[171,172]
*V. angustifolium*	ZBM	Shoot regeneration	Leaf	Ex vitro	[171]
*V. angustifolium* cv. ‘Dwarf Tophat’	WPM	Shoot proliferation	Single node	In vitro on WPM	[173]
*V. angustifolium*	ZBM	Shoot regeneration	Internodes	N/R	[174]
*V. ashei* cv. ‘Titan’	MSM & WPM	Shoot proliferation	Multiple shoots	Ex vitro	[175]
*V. corymbosum* cv. ‘Polaris’, ‘St. Cloud’	MBM-C	Shoot proliferation	AxillaryShoots	Ex vitro	[162]
*V. corymbosum* cv. ‘Huron’	MSM & WPM	Shoot proliferation	Nodal segments	Ex vitro	[176]
Hybrid of *V. corymbosum* ‘Spartan’ × *V. bracteatum*	MSM & WPM	Shoot proliferation	Axillary buds	In vitro	[177]
*V. corymbosum* cv. ‘Berkeley’, ‘Bluecrop’ ‘Goldtraube’	MSM & ANM	Shoot multiplication	Shoots	In vitro on ANM	[178]
*V. corymbosum* cv. ‘Elliot’	WPM	Shoot regeneration and proliferation	Buds, leaves, microshoots	Ex vitro	[179]
*V. corymbosum* cv. ‘Bluecrop’ ‘Berkeley’, ‘Earliblue’	MSM & WPM	Shoot proliferation	Nodal segments	In vitro	[180]
*V. corymbosum* × *V. angustifolium* cv. ‘Northland’	WPM	Shoot regeneration	Nodal and leaf segments	In vitro	[151]
Interspecific hybrids of *Vaccinium* spp.	MSM & ZBM	Shoot regeneration	Ovule	Ex vitro	[181]
*V. corymbosum* cv. ‘Ozarkblue’	WPM	Shoot proliferation and regeneration	Nodal and leaf segments	In vitro & ex vitro	[136]
*V. corymbosum* cv. ‘Bluecrop’, ‘Duke’, ‘Sunrise’.	WPM	Adventitious shoot regeneration	Leaf	Ex vitro	[182,183]
*V. corymbosum* cv. ‘Bluecrop’	WPM	Shoot regeneration	Leaf	Ex vitro	[184]
*V. virgatum* cv. ‘Kunisato 35 Gou’	MSM & WPM	Shoot multiplication	Nodal segments	In vitro	[185]
*V. corymbosum* cv. ‘Berkeley’	WPM	Shoot proliferation	Nodal segments	Ex vitro	[186]
*V. corymbosum* cv. ‘Herbert’	ZBM	Shoot proliferation and regeneration	Nodal segments	Ex vitro	[169]
*V. corymbosum*	WPM	Shoot proliferation	Single node	N/R	[187]
*V. corymbosum* × *V. angustifolium* cv. ‘Northblue’	ZBM	Shoot proliferation	Shoot tips	Ex vitro	[159,160,161]
*V. corymbosum* × *V. angustifolium* cv. ‘North Country’	WPM	Shoot proliferation and regeneration	Leaf segments	N/R	[188]
*V. corymbosum* (southern highbush)	MSM & WPM	Shoot regeneration	Leaf segments	Ex vitro	[189]
*V. macrocarpon* cv. ‘Ben Lear’ ‘Pilgrim’ ‘Stevens’	MBM-C	Shoot proliferation	Nodal segments, shoot tips	In vitro & ex vitro	[46,190]
*V. macrocarpon* wild clones	MBM-C	Shoot proliferation	Nodal segments	In vitro & ex vitro	[190,191]
*V. vitis-idaea ssp. minus* wild clones	MBM-C	Shoot proliferation	Nodal segments	Ex vitro	[192,193]
*V. vitis-idaea* ssp. *vitis-idaea* cv. ‘Regal’, ‘Splendor’ ‘Erntedank’	MBM-C	Shoot proliferation	Nodal segments	Ex vitro	[192,193]
*V. vitis-idaea* ssp. *vitis-idaea* cv. ‘Regal’, ‘Splendor’ ‘Erntedank’	MBM-C	Shoot regeneration	Leaf segments	Ex vitro	[166,194]
*V. myrtillus*	WPM	Shoot proliferation	Auxiliary buds	Ex vitro	[195]

^1^ Media: MBM-C = Modified basal medium for cranberry [46]; MSM = Murashige and Skoog medium [153]; WPM = Woody plant medium [196]; MSM & WPM = 50% MSM and 50% WPM; ZBM = Zimmerman and Broome medium [168]; ANM = Anderson’s Rhododendron medium [150]; N/R = not reported.

**Table 4:** Biochemical and morphological differences among *Vaccinium* berry plants propagated by stem cutting (SC) and tissue culture (TC).

Berry Types	Stem Cutting	Shoot Proliferation	Shoot Regeneration	References
Highbush blueberries	Compared to TC plants, SC plants grow slower, produce less and shorter shoots, greater number of flowers, larger berries and develop flowers a year earlier.	Plants grow faster with taller and more shoots, higher plant dry weight, less flowers and smaller berries than SC plants.	Plants grow faster with taller and more shoots, less flowers and smaller berries than SC plants.	[197,199]
Half-high blueberries	Less and shorter shoots, low berry yield than TC plants.	Grow faster with taller and more shoots and higher fruit yield than SC plants.	-	[198]
Lowbush blueberries	Number of flowers and berries, size and weight berries were greater than TC plant.	Faster vegetative growth with more stems, branches, bigger leaves and larger canopy than SC plants.	-	[30,130,173]
Lingonberries	Higher berry weight, diameter and number per plant than TC plants.	Taller plant, more rhizomes and leaves per plant than SC plants.	Taller plant, more rhizomes and leaves per plant than SC and node culture.	[32,193,202]
Cranberries	Less runners and uprights than TC plants.	More runners, uprights, leaves per upright than the SC plants.	-	[190]
Blueberries	Less phenolic and flavonoid content than TC plant.	Higher contents of phenolics and flavonoids and their antioxidant activity than SC plants.	-	[30,163,203]
Lingonberries	Less total phenolics, anthocyanins, tannins and antioxidant activities than TC plants.	Higher total phenolics, flavonoids, tannins and antioxidant activity in berries of TC plants.	Higher total phenolic flavonoids tannins and antioxidant activity in berries.	[32,195,202]
